# The Evaluation of Plasma and Leukocytic IL-37 Expression in Early Inflammation in Patients with Acute ST-Segment Elevation Myocardial Infarction after PCI

**DOI:** 10.1155/2015/626934

**Published:** 2015-04-16

**Authors:** Xin Wang, Xiangna Cai, Lan Chen, Duanmin Xu, Jilin Li

**Affiliations:** ^1^Department of Cardiology, First Affiliated Hospital of Shantou University Medical College, No. 57, Changping Road, Shantou, Guangdong 515041, China; ^2^Department of Plastic Surgery, First Affiliated Hospital of Shantou University Medical College, No. 57, Changping Road, Shantou, Guangdong 515041, China

## Abstract

*Objective*. Acute ST-segment elevation myocardial infarction (ASTEMI) is accompanied by increased expression of inflammation and decreased expression of anti-inflammation. IL-37 was found to be involved in the atherosclerosis-related diseases and increased in acute coronary syndrome. However, the level of IL-37 in blood plasma and leukocytes from patients with ASTEMI after percutaneous coronary intervention (PCI) has not been explored. *Methods*. We collected peripheral venous blood from consented patients at 12 h, 24 h, and 48 h after PCI and healthy volunteers. Plasma IL-37, IL-18, IL-18-binding protein (BP), and high sensitive C reaction protein (hs-CRP) were quantified by ELISA and leukocytic IL-37 and ICAM-1 by immunoblotting. *Results*. Plasma IL-37, IL-18, and IL-18 BP expression decreased compared to those in healthy volunteers while hs-CRP level was high. Both leukocytic IL-37 and ICAM-1 were highest expressed at 12 h point but significantly decreased at 48 h point. *Conclusion*. These findings suggest L-37 does not play an important role in the systematic inflammatory response but may be involved in leukocytic inflammation in ASTEMI after PCI.

## 1. Introduction

Despite modern reperfusion strategies have been well accepted around the world, acute myocardial infarction (AMI) still remains a leading cause of death worldwide. This suggests that AMI patients still need more understanding of potential pathophysiology for recurrent events of treatment especially in the early post-ACS period. So far, the systemic inflammatory response after AMI has been well described and may play an important role in series of events after AMI. Both circulating inflammatory markers, such as interleukin- (IL-) 6 and high sensitive C reaction protein (hs-CRP), as well as circulating inflammatory cells, including leukocytes and inflammatory monocytes, are elevated acutely after an AMI event in a temporal pattern that corresponds to elevated event rates and is predictive of recurrent events [1].

Anti-inflammation strategy is a good option of improving treatment for patients after AMI. Anti-inflammatory cytokines such as IL-10 are involved in the events of early AMI. The ratio of IL-18/IL-10 is found as an indicator for prognosis of AMI [2, 3]. IL-37 is a recently found anti-inflammatory cytokine in the IL-1 ligand family and proved as a fundamental inhibitor of innate immunity [4]. IL-37 was elevated in some inflammatory diseases such as inflammatory bowel disease, atopic dermatitis, rheumatoid arthritis, and systemic lupus erythematosus [5–8], indicating IL-37 may have potential protective effect on inflammatory diseases. IL-37 is found to be increased in patients with acute coronary syndrome [9] but not investigated in patients after percutaneous coronary intervention (PCI).

Additionally, IL-37 is normally expressed at low levels in peripheral blood mononuclear cells (PBMCs), mainly monocytes, and dendritic cells (DCs) [9], which is rapidly upregulated in the inflammatory context after AMI. IL-37 effectively suppresses the activation of macrophage and DCs [10], and therefore IL-37 may conversely inhibit the production of inflammatory cytokines in PBMCs and DCs after AMI. Given that IL-37 may be associated with the development of atherosclerosis, we hypothesize that IL-37 may play a potential role in the inflammation response including plasma and leukocytes in AMI patients after PCI.

So IL-37, as a new anti-inflammatory cytokine, may suppress immune responses and inflammation [11]. Inflammation is an important step after AMI. Understanding of IL-37 expression helps us further to explore that role and effect of IL-37 in AMI situation. Since it is also reported that a complex of the IL-37 and IL-18-binding protein reduces IL-18 activity [12], we wanted to explore (1) the expression of plasma and leukocytic IL-37 in early period after AMI; (2) the possible relationship between plasma IL-37, IL-18, and IL-18BP; and (3) the possible inhibitory effect of IL-37 on ICAM-1 in leukocytes.

## 2. Methods

### 2.1. Materials

*β*-Actin antibody was purchased from Santa Cruz Biotechnology (Dallas, Texas, USA), and HRP-conjugated rabbit anti-human IgG was purchased from Jackson ImmunoResearch (West Grove, PA, USA); ICAM-1 and IL-18 antibody were purchased from Abcam (Cambridge, MA, USA). IL-37 was purchased from Adipogen AG, Liestal, Switzerland, IL-18 from MBL, Nagoya, Japan, IL-18BP from RayBiotech, Norcross GA, USA, and hs-CRP from Elisa Biotech (Shanghai, China).

### 2.2. Patients Population

From October, 2013, to April, 2014, a total of 112 cases of healthy volunteers (56) and patients (56) with ASTEMI agreed to participate in this test. STEMI was defined as chest pain suggestive of myocardial ischemia for at least 30 minutes before hospital admission and the electrocardiogram (ECG) with new ST-segment elevation in 2 or more contiguous leads of 0.2 mV or more in leads V2 to V3 and/or 0.1 mV or more in other leads. The exclusion criteria were as follows: (1) patients presenting with STEMI after 12 hours from symptom onset; (2) patients presenting with vasospastic angina (as determined by the resolution of ST-segment elevation and relief of symptoms after an IV administration of nitroglycerin); (3) patients over 75 years old; (4) recent (<1 week) systemic or local inflammation disease; (5) organ (liver, kidney) dysfunction; (6) cardiogenic shock.

Emergency PCI procedure must be carried out on all patients within 2 hours. All patients will receive 300 mg aspirin and a loading dose of 600 mg clopidogrel before the procedure. Unfractionated heparin will be administered intravenously in boluses to maintain an activated clotting time of >250 seconds during the procedure. Administration of glycoprotein IIb/IIIa inhibitors will be based on the physicians' discretion. PCI will be performed according to current international guidelines. The goals of the procedure are to achieve optimal angiographic efficacy of PCI at the infarct-related artery and minimize the risk of procedure-related complications. A full range of commercially available guiding catheters, balloon catheters, and guide wires will be readily available. All patients included in this trial will be treated according to the current American College of Cardiology (ACC)/American Heart Association (AHA) guidelines regarding poststenting management, which specify treatment with at least 100 mg of aspirin daily and 75 mg clopidogrel daily for at least 12 months after PCI. Angiotensin converting enzyme inhibitors and *β*-blockers will be administrated after PCI if no limitation exists.

### 2.3. Leukocytes and Blood Plasma Isolation

Blood collection from consented healthy volunteers and patients was approved by the Human Ethics Committee of First Affiliated Hospital of Shantou University Medical College. An approximate volume of 3 mL peripheral venous blood was collected from all patients at different time point (12 h, 24 h, and 48 h) after PCI procedure into the procoagulation tube and ethylenediaminetetra acetic acid (EDTA)-K2 anticoagulation tube separately. Sample collection from healthy volunteers was obtained in the morning. Plasma was isolated from blood sample in procoagulation tube after centrifugation at the speed of 3,000 rpm, 15 min. Leukocyte was isolated from blood sample in EDTA-K2 tube using erythrocyte lysis buffer (Qiagen, Hilden, Germany) and then collected after centrifugation at the speed of 2000 r/min, 10 min. Leukocytic protein was stored at −80°C for immunoblotting.

### 2.4. Immunoblotting

Immunoblotting was used to detect leukocytic IL-37 and ICAM-1. Samples were separated on 10% SDS-polyacrylamide gels and transferred onto nitrocellulose membranes. Membranes were blocked for 1 h at room temperature with 5% dry milk in TPBS (PBS containing 0.1% Tween 20) and then incubated with the appropriate primary antibodies (ICAM-1 antibody was diluted into 1 : 1000, IL-37 1 : 500, and *β*-blocker 1 : 1000) overnight at 4°C. After washing with TPBS, membranes were incubated with horseradish peroxidase- (HRP-) linked secondary antibodies (1 : 5000 dilution with TPBS containing 5% dry milk) at room temperature for 1 h. Bands were developed using ECL and exposed on X-ray films. Band density was analyzed using NIH ImageJ software.

### 2.5. Elisa

Plasma hs-CRP, IL-18, IL-18BP, and IL-37 were quantified by ELISA kits. Plasma hs-CRP was just detected at 12 h point, while IL-18, IL-18BP, and IL-37 were detected at 12 h, 24 h, and 48 h point. Recombinant cytokines were used to construct standard curves. Absorbance of standards and samples was determined spectrophotometrically at 450 nm using a microplate reader (KHB labsystem wallscan k3, Thermo Scientific, Finland). Results were plotted against the standard curve. The assays were carried out according to the protocols provided by the manufacturer.

### 2.6. Statistic Analysis

Data are expressed as mean ± standard error of mean (SEM). Analysis of variance (ANOVA) was performed, and differences were considered significant when *P* < 0.05, as verified by Fisher post hoc test.

## 3. Results

### 3.1. Baseline Characteristics

Baseline clinical characteristics on the basis of age, gender, and leukocyte count between healthy volunteers and patients with STEMI are presented in Table 1. Mean age, gender disturbance, and leukocyte count were balanced between the groups. Most patients have 2 or more cardiovascular risk factors, while few patients have single parameter (2 have only hypertension, 2 only diabetes, 4 only hypercholesterolemia, and 4 only smoking history). Most patients have one or two diseased arteries, while 3 cases have 3 diseased ones. And most patients were occluded in LAD or RCA, while only 3 cases were infarcted in LCX. Plasma hs-CRP was elevated and indicated high inflammation situation after AMI with PCI.

### 3.2. Plasma IL-37, IL-18, and IL-18BP Decreased at Early Period

We tested plasma IL-37, IL-18, and IL-18BP expression at different time points (12 h, 24 h, and 48 h after PCI) in patients with STEMI with Elisa kit (Table 2). Both IL-37, IL-18, and IL-18BP expression were balanced but decreased at all time points in patients if compared to those in healthy volunteers (*P* < 0.05). We then test IL-37 and ICAM-1 expression in leukocytes to check cellular inflammation and anti-inflammation after PCI.

### 3.3. Leukocytic IL-37 and ICAM-1 Expression Change

**Figure 1:**
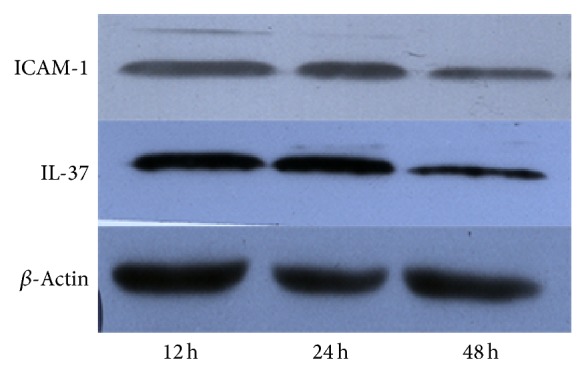
Leukocytic IL-37 and ICAM-1 expression change at early period. Leukocytic IL-37 and ICAM-1 protein expression were detected with immunoblotting. Both leukocytic IL-37 and ICAM-1 protein were highest expressed at 12 h point but significantly decreased at 48 h point (*P* < 0.05, *n* = 48).

Leukocyte is an important cell involved in inflammatory response after AMI and expressed IL-37 under inflammation [4]. We analyzed cellular IL-37 and ICAM-1 protein expression with immunoblotting. We found that both cellular IL-37 and ICAM-1 protein were highest expressed at 12 h point but significantly decreased at 48 h point (Figure 1, *P* < 0.05).

## 4. Discussion

In this study, we demonstrated the expression of plasma IL-37 and leukocytic IL-37 in STEMI patients after PCI in early period. We found that plasma IL-37 did not increase and leukocytic IL-37 went down while plasma hs-CRP is high which indicates high inflammation response in the first 2 days after PCI. Plasma IL-18, which was found to be inhibited by IL-37, was also not increased under this situation.

STEMI is usually associated with inflammation and develops into severe complications. It is proved that inflammation is involved in atherosclerostic plaque formation and rupture, coronary thrombosis, and myocardial necrosis and repair after myocardial infarction [13–15]. Anti-inflammation strategy may be good for myocardial prognosis, but some anti-inflammatory cytokines such as IL-10 reduced in ACS patients, reflecting the imbalance in systemic cytokine response following an ACS [2, 16]. IL-37 is already proved as an anti-inflammatory cytokine and reported to be elevated in ACS patients [9], but we found that it decreased in patients after PCI in our study. This may be because PCI treatment could inhibit systematic IL-37 expression. Hereby, we found that systematic plasma IL-37 and leukocytic IL-37 decreased in the early period. In vivo expression of human IL-37 in mice reduces local and systemic inflammation in ConA-induced hepatitis and LPS challenge [17]. Therefore, IL-37 reduction may fail to inhibit systematic inflammation in patients with ASTEMI after PCI.

Not only hs-CRP but also IL-18 had been proved to be good indicators for prognosis for patients [18–20]. IL-18 is enhanced in STEMI situation [21–23], and the expression change of IL-18 is not reported before in patients after PCI. We found that IL-18 is decreased in the patients with PCI compared to that in healthy volunteers. A complex of the IL-37 and IL-18-binding protein reduces IL-18 activity [12]; but plasma IL-37, IL-18 and IL-18BP were increased in patients with ACS [9]. We wanted to test whether there is any relationship between changes of IL-18 and IL-37 in STEMI after PCI. The synchronous reduction of systematic IL-37 and IL-18 could not reveal the inhibitory effect of IL-37 on IL-18. The reduction of systematic IL-18 after PCI is not due to enhanced anti-inflammatory response. The ratio of IL-18/IL-10 was found to be an independent predictor of adverse events in patients with ACS [2, 3]. To decrease IL-18 and increase IL-10 are helpful for the recovery of patients with ACS [2]. The potential predictor effect of the ratio of IL-18/IL-37 on adverse events could be explored in the future. And to change the imbalance between inflammation and anti-inflammation is still meaningful. Although inflammatory markers such as CRP predict future cardiovascular events in ACS patients, when all inflammatory mediators are taken into account in a prospective analysis of risk, markers reflecting anti-inflammatory mechanisms may be better prognostic markers [18]. Furthermore, elevated level of plasma IL-37, IL-18, and IL-18BP had no correlation with the severity of the coronary artery stenosis [9], and decreased level of those was not related with that in our study.

Leukocyte is an important inflammatory cell in STEMI patients and is involved in myocardial necrosis and repair after STEMI [24]. Circulating monocytes could express high level of proinflammatory cytokines, TNF-alpha, and IL-6, as well as anti-inflammatory cytokine IL-10 [25]. ICAM-1 induces the interaction between leukocytes and endothelial cells [26], which is involved in the myocardial remodeling. We isolated circulating leukocytes and found reduction of both ICAM-1 and IL-37 in early period, indicating a balance of inflammatory and anti-inflammatory response in circulating leukocytes. We can suggest that ICAM-1 expression decreased because of IL-37, which is better to be confirmed by isolated leukocyte culture. Different from the systematic imbalance, the changes of IL-37 and ICAM-1 indicated a balance of inflammation and anti-inflammation on leukocytes. Leukocytes include several types including neutrophils, lymphocytes, and monocytes, so we cannot acutely tell which subtype has only or more IL-37 expression. We think neutrophils, most percentage of leukocytes, may be a potential good target to explore IL-37 expression and change in the future.

In this study, our baseline is healthy volunteers without some cardiovascular risk factors except age and gender. There is no report about systematic IL-37 expression in healthy volunteers before. We found that it is lower in the STEMI than that in the healthy, which may be because anti-inflammatory response is inhibited [2, 16]. Compared to other studies about anti-inflammatory cytokines, we can suggest that this is due to the inhibitory effect of anti-inflammatory response in ACS. Enhancing anti-inflammatory effect in STEMI may help to repair myocardial damage [27, 28]. How to increase IL-37 expression may be helpful and it is interesting to investigate that in future study. Furthermore, sample size is not too much in our study; we can investigate expression difference in different subgroup if we have more samples.

## 5. Conclusion

In conclusion, our study firstly demonstrates that systematic IL-37 expression was decreased in STEMI with PCI situation and on decline in leukocytes after PCI. These suggest that IL-37 does not play an important role in the systematic inflammatory response but may be involved in leukocytic inflammation in ASTEMI after PCI. More studies should be investigated for that.

## Figures and Tables

**Table 1:** Baseline characteristics of healthy volunteers and patients with ASTEMI after PCI.

	Patients (*n* = 56)	Healthy populations (*n* = 56)	*P* value
Age (years)	56.5 ± 1.82	56.7 ± 2.22	0.532
Male gender	46	40	0.605
WBC (∗10E9/L)	9.48 ± 0.62	8.65 ± 0.45	0.876
Hs-CRP (mg/L)	24.98 ± 3.33		
Cardiovascular risk factors			
Hypertension	28		
Diabetes mellitus	16		
Hypercholesterolemia	30		
Smoking history	30		
Ischemic time (min)			
Mean	385		
Median	245		
Number of diseased vessels			
1	24		
2	23		
3	6		
Infarct-related artery			
LAD	34		
LCX	3		
RCA	19		

WBC: white blood cells; LAD: left anterior descending artery; LCX: left circumflex artery; RCA: right coronary.

**Table 2:** Plasma IL-37, IL-18, and IL-18BP expression decreased in 48 h after PCI procedure.

Group	Patients (*n* = 56)			Healthy (*n* = 56)
	12 h	24 h	48 h	
IL-37 (pg/mL)	82.8 ± 14.79^*^	82.2 ± 9.28^*^	84.4 ± 13.35^*^	120.6 ± 2.67
IL-18 (pg/mL)	46.9 ± 5.06^*^	44.2 ± 5.28^*^	43.1 ± 4.60^*^	91.0 ± 2.80
IL-18BP (pg/mL)	231.9 ± 22.06^*^	261.5 ± 24.18^*^	234.6 ± 19.53^*^	461.9 ± 62.06

Plasma IL-37, IL-18, and IL-18BP from healthy volunteers and patients at different time point were qualified by Elisa kit. Lower expression of both IL-37, IL-18, and IL-18BP was in patients compared to those in healthy volunteers (^∗^ *P* < 0.05). No difference of change in all plasma cytokines was expressed at different time point in the patients with ASTEMI after PCI.

## Abbreviations

ACS: Acute coronary syndrome
AMI: Acute myocardial infarction
DC: Dendritic cell
Hs-CRP: High sensitive C reaction protein
ICAM-1: Intercellular adhesion molecule-1
IL: Interleukin
LPS: Lipopolysaccharide
PBMC: Peripheral blood mononuclear cell
PCI: Percutance coronary intervention.
